# Characteristic analysis of Omicron‐included SARS‐CoV‐2 variants of concern

**DOI:** 10.1002/mco2.129

**Published:** 2022-04-09

**Authors:** Hao Yang, Penghui Liu, Yong Zhang, Tingfu Du, Yanan Zhou, Shuaiyao Lu, Xiaozhong Peng

**Affiliations:** ^1^ Institute of Medical Biology Chinese Academy of Medical Sciences and Peking Union Medical College Kunming Yunnan China; ^2^ State Key Laboratory of Medical Molecular Biology Department of Molecular Biology and Biochemistry Institute of Basic Medical Sciences Medical Primate Research Center Neuroscience Center Chinese Academy of Medical Sciences School of Basic Medicine Peking Union Medical College Beijing China

**Keywords:** ACE2 binding, Omicron, SARS‐CoV‐2, spike protein, variants of concern

## Abstract

In view of the rapid development of the COVID‐19 pandemic and SARS‐CoV‐2 mutation, we characterized the emerging SARS‐CoV‐2 variants of concern (VOCs) by both bioinformatics methods and experiments. The representative genomic sequences of SARS‐CoV‐2 VOCs were first downloaded from NCBI, including the prototypic strain, Alpha (B.1.1.7) strain, Beta (B.1.351) strain, Delta (B.1.617.2), and Omicron (B1.1.529) strain. Bioinformatics analysis revealed that the D614G mutation led to formation of a protruding spike (S) in the tertiary structure of spike protein, which could be responsible for the enhanced binding to angiotensin‐converting enzyme 2 (ACE2) receptor. The epitope analysis further showed that the S protein antigenicity of the Omicron variant changed dramatically, which was possibly associated with its enhanced ability of immune escape. To verify the bioinformatics results, we performed experiments of pseudovirus infection and protein affinity assay. Notably, we found that the spike protein of Omicron variant showed the weakest infectivity and binding ability among all tested strains. Finally, we also proved this through virus infection experiments, and found that the cytotoxicity of Omicron seems to be not strong enough. The results in this study provide guidelines for prevention and control of COVID‐19.

## Graphical abstract

In this study, we first predicted and compared the structure of the S protein and B‐cell epitopes of different SARS‐CoV‐2 variants. Then, the binding ability of different SARS‐CoV‐2 variant S proteins to angiotensin‐converting enzyme 2 (ACE2) cells and the affinity of RBD region to ACE2 were further compared through pseudovirus infection and intermolecular binding ability test. Finally, cell infection experiments were performed. The results unexpectedly showed that Omicron possesses lower ACE2 binding capacity, and lower replication capacity than Delta strain.

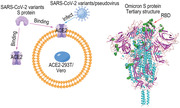

## 1 INTRODUCTION

On January 30, 2020, SARS‐CoV‐2 was designated a public health emergency of international concern, and the World Health Organization declared it a pandemic on March 11, 2020. As of February 7, 2022, nearly 387 million people had been infected, and more than 5.7 million people had died.^1^ SARS‐CoV‐2 belongs to the coronavirus family. It is a single‐sense‐sense RNA (+ssRNA) virus.^2^ SARS‐CoV‐2 is unique among the known β‐coronaviruses, because it incorporates multiple cleavage sites, which is currently known to increase pathogenicity and transmission in other viruses.^3^ In July 2020, it was reported that the strain with the spike protein D614G mutation in Europe is more contagious and may become the main form of the virus pandemic.^4^ In December 2020, the United Kingdom (UK) reported a new SARS‐CoV‐2 variant of concern (VOC), classified as pangolin lineage B.1.1.7 (Alpha variant^2^). Soon thereafter, there was a rapid increase in infections in the UK and other regions. Subsequently, the B.1.351 (Beta variant) variant was reported in South Africa.^5^ In addition, India has also seen variants such as B.1.617 and B.1.618. Recently, the daughter B.1.617, B.1.617.2 (Delta variant), has spread worldwide at a higher speed.^6^ A new SARS‐CoV‐2 variant Omicron was recently reported in South Africa, which has 32 mutations in the spike protein alone.^7^ And in a short period of time, the variant has now spread rapidly to many countries and regions, The Omicron variant may be more contagious than the Delta and Beta variants.^8^ It is expected to replace Delta as a new trend in the pandemic.

The SARS‐CoV‐2 genome is about 30 kb in length, including ORF1a and ORF1b, fragments for four structural proteins and several accessory proteins.^9^ There is a 76‐base leader sequence (TRS) at the 5′ end of its RNA, which also exists near each ORF to regulate the discontinuous synthesis of the negative strand in the middle of the sgRNA.^10^ Like other coronaviruses, ORF1a and ORF1ab inside the host cell are converted into the polyproteins pp1a and pp1a/b. These polyproteins generate 16 nonstructural proteins (Nsp) by proteolytic cleavage of nsp3 (papain‐like protease) and nsp5 (major protease). Nsp12 (RdRP) and its cofactors Nsp7 and Nsp8 form a replication–transcription complex, which is used to further synthesize genome and sgRNA for translation of structural and accessory proteins.^9^ SARS‐CoV‐2 has four structural proteins, namely S (spike), E (envelope), M (membrane), and N (nucleocapsid) proteins. The N protein wraps the RNA genome, while the S, E, and M proteins together form the viral envelope.^9^ The S protein is responsible for attaching the virus to and fusing with the host cell membrane.^11^ Specifically, the S1 subunit of S protein catalyzes viral attachment to the host cell, while the S2 subunit catalyzes fusion with the cellular membrane. Modeling experiments of the viral spike protein show that SARS‐CoV‐2 has sufficient affinity for the angiotensin‐converting enzyme 2 (ACE2) receptor on human cells. It can be used as a mechanism for virus entry into cells.^12^

The crystal structure of RBD in complex with ACE2 indicates the molecular mechanism of the initial steps of SARS‐CoV‐2 infection.^13^ The SARS‐CoV‐2 RBD is located between the S1 subunit and the N‐terminal domain (NTD) of the spike protein and consists of approximately 200 amino acids. It interacts with ACE2 through a receptor binding motif, which contains approximately 70 amino acids, 17 of which are responsible for direct contact with ACE2.^13^ It has been reported that SARS‐CoV‐2 VOCs (Alpha, Beta, Delta, and Omicron) alter their affinity to ACE2 and the ability to escape immunity to varying degrees, but do not alter the overall structure of the S protein.^14^ For example, S protein of the D614G variants has a looser and wider trimer structure of RBD.^15^ And compared to the original strain of SARS‐CoV‐2, the D614G mutation makes the S protein more stable and more flexible, which makes the virus more infectious.^16^, ^17^, ^18^ As a key determinant of host membrane fusion, the S protein has become the key factor in the study of SARS‐CoV‐2 infection mechanisms and a major target for the development of therapeutic antibody, as well as a major immunogen in humans. A few potent neutralizing mAbs have been shown to effectively inhibit viral binding to the host receptor ACE2 in vitro and in vivo. The mixtures of these neutralizing mAbs targeting noncompeting epitopes may improve the efficacy of Ab‐based therapies and prevent the emergence of SARS‐CoV‐2 immune‐escaping variants to some extent.^19^

SARS‐CoV‐2 may undergo new mutations over time, and these mutations may further enhance the spread and pathogenicity of the virus. Therefore, it is necessary to pay attention to the characteristics of newly emerged variants viruses in time and take timely measures. This study reports the tertiary structure characteristics and differences of the S protein of different SARS‐CoV‐2 variants and predicts the trend of epitope changes between different strains. The difference in infection ability and ACE2 binding ability between different variants was proved by experiments.

## 2 RESULTS

### 2.1 The changes in structure of the S protein of VOCs predictively facilitates entry of the virus into the host cell

**FIGURE 1:**
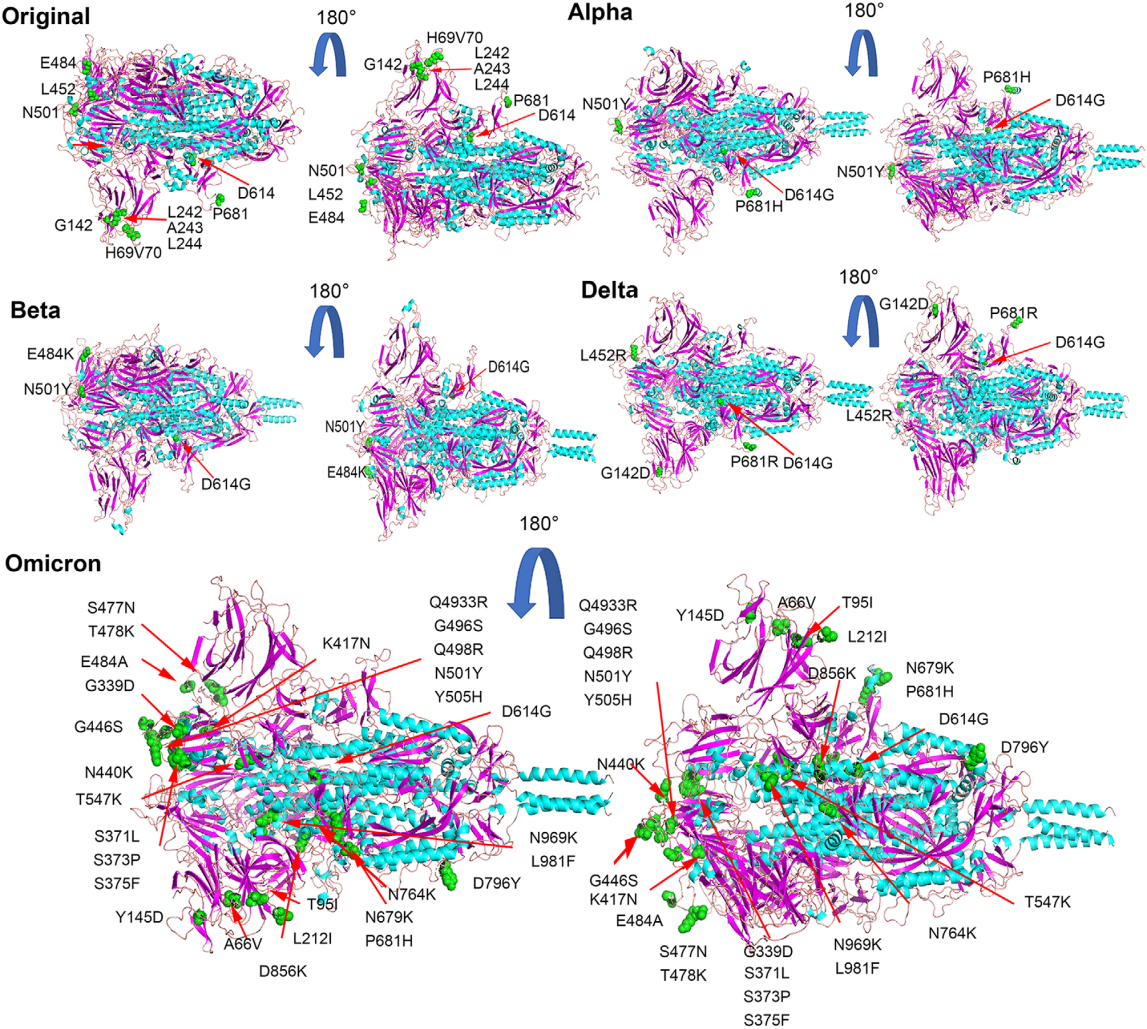
Prediction of the three‐dimensional structure of spike proteins. The tertiary structure of the spike protein of the five strains was predicted through the Swiss‐Mode online platform, and the spike protein was rendered and annotated by PyMOL software. As shown, the mutations in other strains or deletions of amino acid residues (green) have been marked, and some key amino acid residue mutations (green) are also marked in different strains

To compare the S proteins of SARS‐CoV‐2 variants, we first modeled the tertiary structures of their S proteins by SWISS‐MODE (Figure 1), and evaluated the reliability of the predicted results by the sequence coverage. Although the tertiary structure of the S protein predicted by our prototype (Template 7cn8.1. A Coverage 91.6%) has no protruding structure with the virion, the actual cryo‐EM revealed a protruding structure similar to that of the variants,^11^ which may be caused by a certain difference between the calculation results of the prediction system and the actual protein folding process, or may be caused by the differences in the template itself. We also compared the S protein structure of the published variants, and the Alpha strain (Template 7n1u.1.A Coverage 100%) was in complete agreement with the one reported.^20^ The mutation in Alpha variant did not cause major structural changes in RBD and NTD. The structure of the S protein of Beta variant (Template 7n1q.1 Coverage 99.92%) is mostly consistent with the report,^20^ and according to the predicted results Delta (Template 7sbo.1 Coverage 99.92%) and Omicron (Template 7to4.1 Coverage 99.84%) are also basically the same as reported.^21^ According to model prediction results and reported literatures, we found that the spike proteins of the four strains are roughly similar in structure, but there are some mutations in the S protein of the Beta, Alpha, Delta, and Omicron strains. These mutations may promote the binding of the spike protein to the receptor and make this binding stronger (Figure 1).^21^, ^22^, ^23^ Compared with S protein of the original strain, there is a prominent loose structure in the S proteins of Alpha, Beta, Delta, and Omicron (Figure 1). Consequently, the RBD regions of Alpha, Beta, Delta, and Omicron strains were more exposed. In addition, Alpha, Beta, Delta, and Omicron strains had partial protruding structures at the junction of S protein and virus particles (Figure 1), which may be caused by the internal extrusion of the S protein after the D614G mutation. This structure may be beneficial for S protein binding to host cell ACE2 receptor by increasing the shake of S protein.^24^ P681H mutation in the Alpha and Omicron strains and P681R mutation in the Delta strain reduced the acidity of the amino acids, thereby improved furin recognition and the digesting efficiency,^25^ which means that more virus particles will enter the host cell.

### 2.2 Differences of antigenic epitopes among variants

**FIGURE 2:**
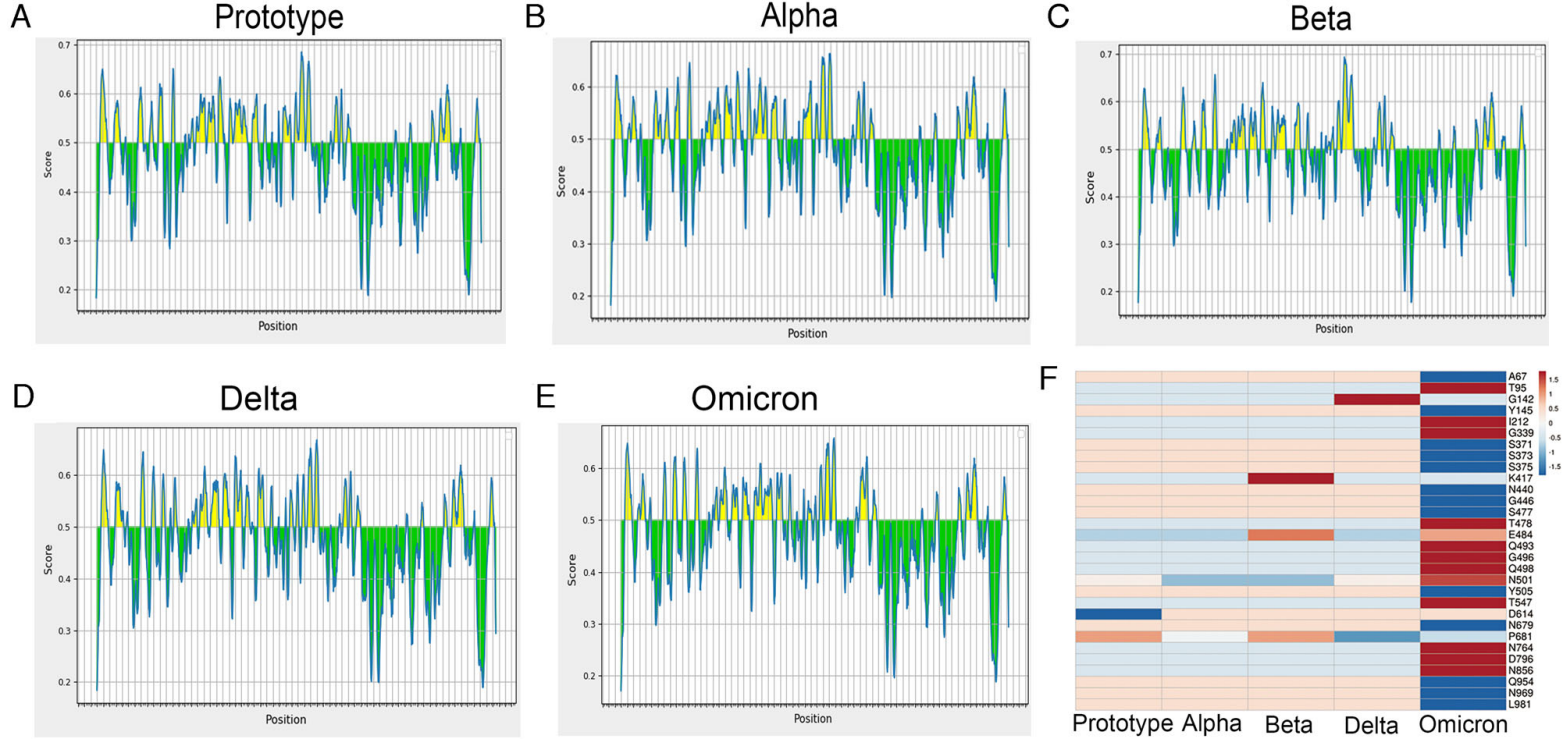
Epitope prediction of the spike protein. Epitope prediction and scoring were performed by the IEBD (https://www.iedb.org/) online program, and the score of a single amino acid was predicted by loading the full amino acid sequence of the S protein of different variants, and the scoring threshold was 0.5. (A) Prototype strain; (B) Beta strain; (C) Alpha strain; (D) Delta strain; (E) Omicron strain; (F) changes in the score of mutation amino acid epitope of spike protein

To compare the B‐cell epitopes in S proteins of different SARS‐CoV‐2 variants, we used the IEBD online program (https://www.iedb.org/), by loading the amino acid sequences of different variant S proteins. We obtained data for different variant S protein epitopes (Figure 2). Our results show that the epitope distribution of different variants is relatively similar, with only some subtle differences (Figure 2A–E), so we further analyzed the antigenic changes of different strains after amino acid site mutations influence. Beta strains L241, A242, and L243 cannot be used as epitopes, so their deletion may not affect their antigenicity. The K417N and D614G epitope scores have almost no change, indicating that the D614G mutation is more about changing the structure of the S protein to promote the spread of the virus. The scores of E484K and N501Y are also relatively close, indicating that these mutations do not affect the epitope of the spike protein of the Beta strain (Figure 2E). In the Alpha strain, there are two key deletions of H60V70 on the epitope, which may affect the immune escape of the S protein (Figure 2E). The P681H mutation slightly reduces the epitope score and may have a certain impact antigenicity (Figure 2E). In the Delta strain, the L452R mutation seems to enhance antigenicity, whereas G142D has no effect on the epitope, but the P681R mutation seems to cause a slight decrease in the antigen score, which may have a certain effect on the epitope (Figure 2E). There are many amino acid deletions and mutations in the Omicron variants. Among them, the scores of T95I and L212I on the NTD have increased slightly, but the changes are not obvious. The same G339D, T478K, Q493R, G496S, and Q498R in the RBD region have also increased the antigen score, but these changes were not significant. While the scores of A67V, Y145D, and N440K on NTD and RBD decreased more, the scores of S371L, S373P, S375F, G446S, and S447N on RBD only slightly decreased (Figure 2E). In general, different mutations in each variant have some effect on the epitope, but the effect of a single mutation on antigenicity does not seem to be decisive. It may be that its effect on viral infection and pathogenicity requires multiple mutations to work together.

### 2.3 Analysis of infection ability and binding ability of S protein to ACE2 of different variants

**FIGURE 3:**
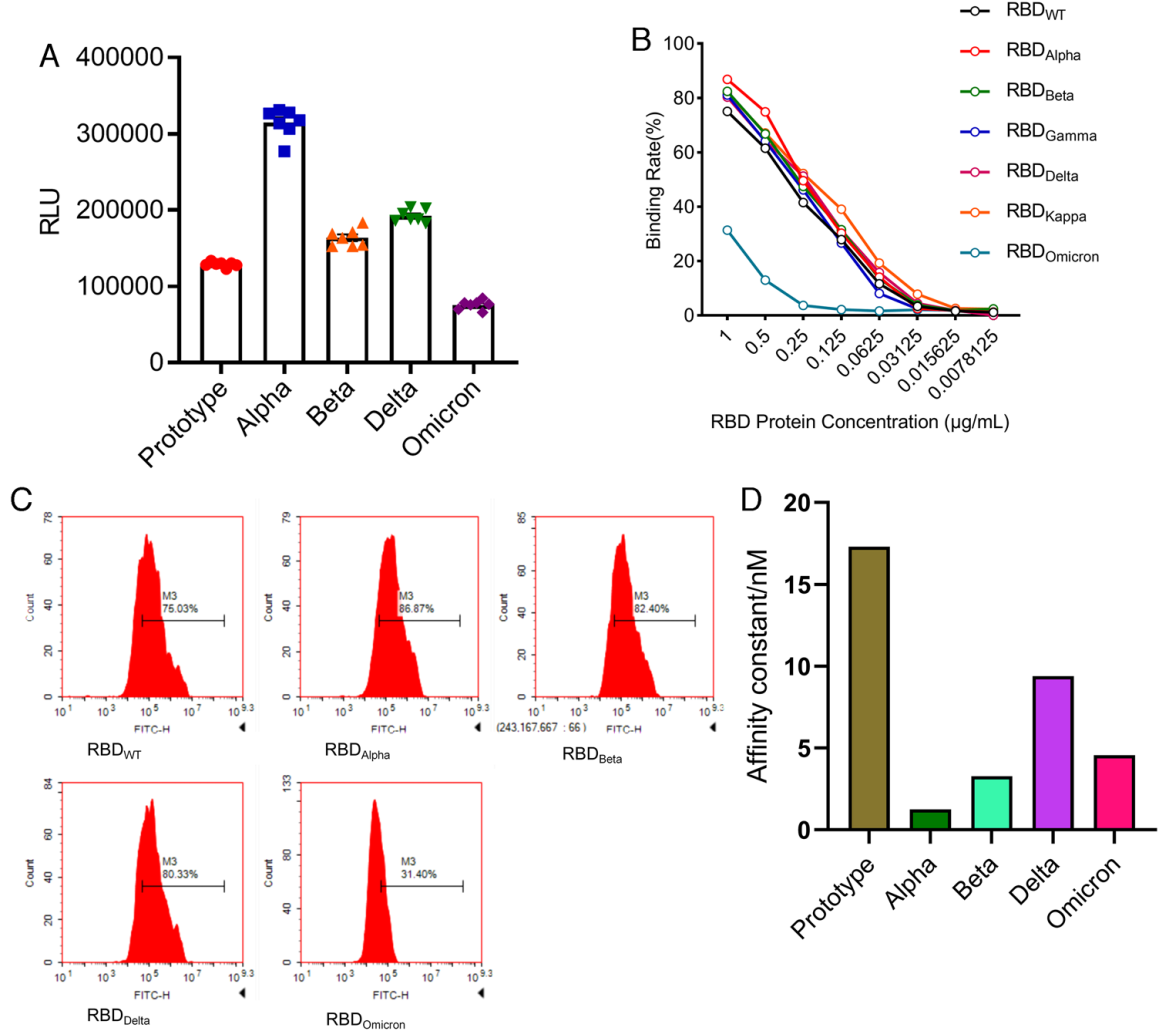
Comparison of infection abilities among SARS‐CoV‐2 VOCs. (A) 293T/angiotensin‐converting enzyme 2 (ACE2) cells were infected by variants of concern (VOCs) pseudoviruses for luciferase assay. (B and C) RBD‐hFc proteins at different concentrations were added to 293T/ACE2 cells. Then hFC flow antibody was added to detect the ratio of cells bound to RBD‐hFC protein. (D) ACE2–Fc was captured on sensor chip protein A. RBD protein was run across the chip, elute each sample by running buffer, and the affinity value (*K*_d_) was determined. The higher the affinity value (*K*_d_), the lower the affinity with ACE2

To understand the infectivity of SARS‐CoV‐2 VOCs, we used the pseudovirus system to evaluate the infectivity of pseudoviruses with different variants S proteins on ACE2 293T cells by luciferase assay. All variant strains except Omicron pseudovirus were found to have stronger infectivity than the prototype strain, and Alpha pseudovirus had the strongest infectivity. This was followed by the Delta strain and the Beta strain, whereas the Omicron strain, which recently attracted attention and became an epidemic, was the least infective pseudovirus, even lower than the prototype strain (Figure 3A). Among the many variant pseudoviruses, Omicron does not appear to be dominant in terms of infectivity. It may be its unique immune evasion ability that makes it become a pandemic. To further confirm the binding ability of S protein of different variants to ACE2‐expressing cells, we performed flow cytometry experiments. We tested the ability of different variants RBD‐hFc proteins to bind to 293T highly expressing ACE2 cells (293T/ACE2) at different concentrations (Figure 3B) and the binding ratio of RBD to 293T/ACE2 cells at a concentration of 1 μg/ml (Figure 3C). The results were consistent with the results of pseudovirus infection, when different concentrations of SARS‐CoV‐2 variant RBD proteins were used to bind to ACE2 cells. The Omicron RBD region still showed extremely low ACE2 binding ability. In the presence of high concentration of RBD, the binding ability of Alpha, Beta, and Delta to ACE2 cells was stronger than that of WT strain, but when the concentration of RBD decreased to 0.0625 μg/ml, this trend was not obvious (Figure 3B,C). To further validate the ability of different SARS‐CoV‐2 variant S proteins to bind to ACE2 in vitro, we evaluated the binding ability of S protein of different variants to ACE2 by intermolecular binding ability experiment. The results showed that the binding ability of S protein of each variant to ACE2 was stronger than that of the prototype. The Delta strain S bind ACE2 ability is the strongest, followed by the Alpha strain and the Beta strain, whereas the Omicron strain has the weakest binding ability of the S protein to ACE2 among the variants (Figure 3D). These may explain the lower ACE2 cell infectivity of the S protein of the Omicron strain (Figure 3A,D). Together, these results show that the affinity of Omicron S protein to ACE2 is weaker than those of the prototypic strain and other variants. Although these results cannot represent the actual virus infection, they can still be used as a reference.

### 2.4 Infection ability of Omicron does not appear to be dominant

**FIGURE 4:**
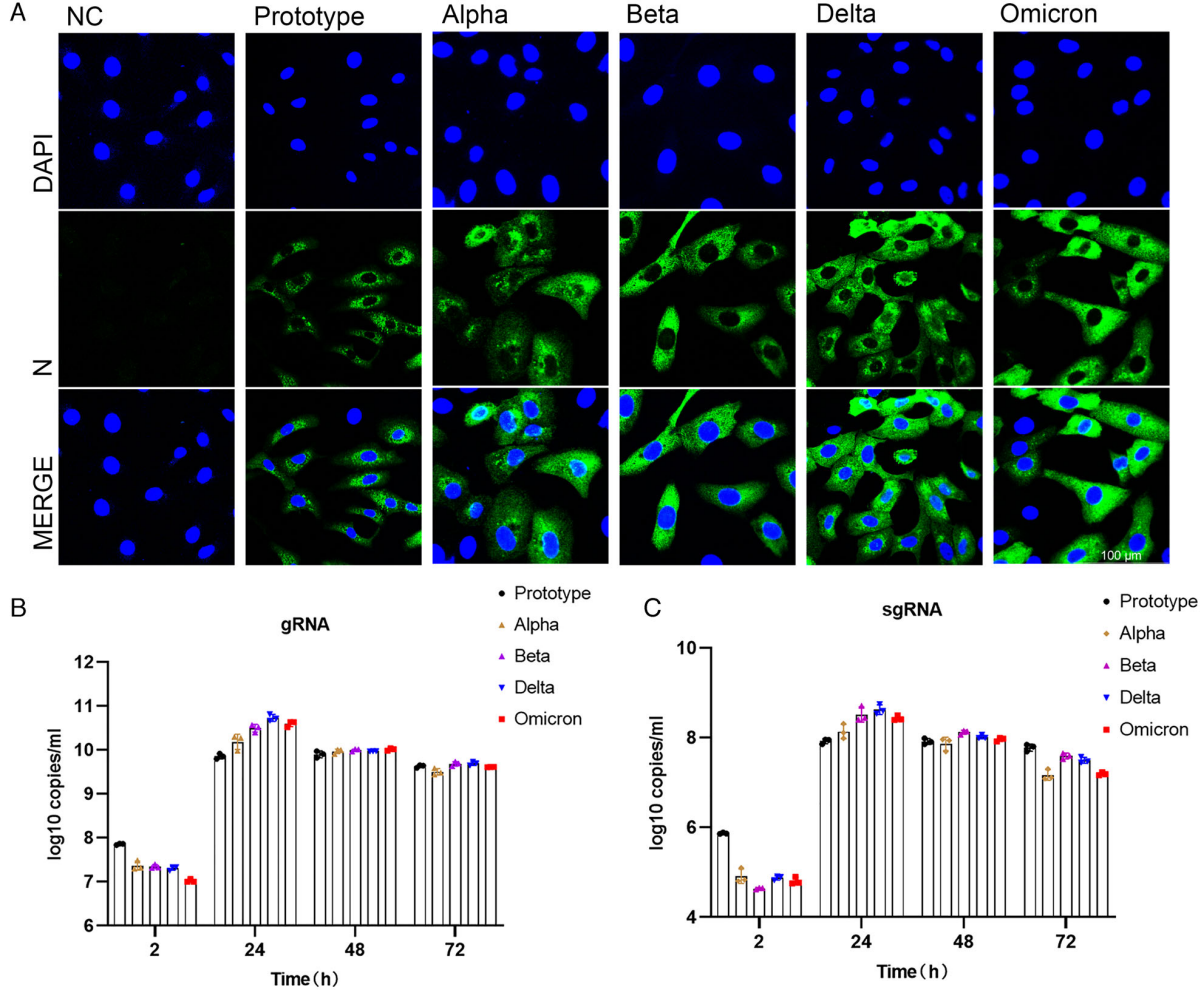
Analysis of Vero cells infected with different SARS‐CoV‐2 variants. (A) Immunofluorescence analysis of viral antigen of different variants in Vero cells 24 h after virus infection. Viral antigen was detected with anti‐N protein antibody. Shot under a 60× confocal microscope. (B) Copy numbers of viral genomes of different variants were measured with N gene by qPCR at 2, 24, 48, and 72 h post virus infection. (C) Subgenome copy number of different variants analyzed by E‐gene via qPCR at 2, 24, 48, and 72 h post virus infection

To further confirm infection ability of VOCs, we performed infection experiments of authentic viruses in Vero cells. Immunofluorescence analysis was conducted for detection of viral nucleoprotein via anti‐N protein antibody at 24 h after virus infection (Figure 4A; Figure S1). The results showed that after 24 h of infection, nucleoprotein was detectable in cells infected with all tested strains, suggesting SARS‐CoV‐2 and its VOCs could replicate and pack in the host cells. However, the prototypic strain showed the lowest level of nucleoprotein among all analyzed strains, and no obvious difference was observed among four VOCs. Next, we harvested both cells and supernatants at different time points post infection and quantitated the copy number of viral genome by qPCR (Figure 4B). We found the highest copy number of viral genome in prototype‐infected cells, the lowest in Omicron, and no obvious difference among the other three variants at 2 h after infection. These results indicated that the binding of Omicron S protein to cellular ACE2 was not dominant, which was partly consistent with results in the pseudoviruses experiment (Figure 3). Then the Delta variant reached the highest copy number of RNA genome at 24 h post infection, and the prototype genome had the lowest copy number. Finally, no obvious difference was observed among all tested strains at 48 and 72 h post infection. Viral E gene was further analyzed via PCR to evaluate the replication ability of the virus (Figure 4C). The results showed that the copy number of the prototype subgenome was also the highest at 2 h after infection, and the copy number of the Omicron subgenome was lower than Delta but higher than Beta. At 24 h post infection, Delta had the highest subgenomic copy number, followed by Omicron. These results indicated that infectivity of Omicron might not be dominant among SARS‐CoV‐2 variants, and its replication ability seems to be second only to Delta. Finally, we compared cytopathic effects (CPE) of prototype and its VOCs on the cells at different time points post viral infection (Figure S2). No CPE appeared at 2 and 24 h post infection. At 48 h, all strains except Omicron showed CPE, and at 72 h, all strains including Omicron showed obvious CPE. However, CPE caused by Omicron was less severe than those of the other strains, suggesting that the cytotoxicity of Omicron may be weaker than other strains.

## 3 DISCUSSION

SARS‐CoV‐2 poses an unprecedented challenge to the health of people around the world and to the global economy. According to data, 220 million novel coronavirus infections have been confirmed worldwide, bringing the cumulative global death toll to 4.59 million at the time of publication. The S protein of SARS‐COV‐2 plays a key role in the infection process of the body. It has been reported that the S protein binds with ACE2 and TMPRSS2 receptors on the surface of the body to assist the virus in entering the body and causing infection. Therefore, the S protein of SARS‐CoV‐2 has been the focus of attention. The main mutation sites of SARS‐CoV‐2 variants are also concentrated on the sequence of the S protein.

From the perspective of bioinformatics, this study interprets the characteristics of the existing widely prevalent SARS‐CoV‐2 variants spike protein (S). In summary, our results indicate that mutations resulted in changes in the tertiary structure of the S protein of different variants. The RBD regions of Alpha, Beta, Delta, and Omicron strains are more exposed, and the junction between the variants S protein and the virion has a prominent structure, which may be caused by the D614G mutation. However, the epitope difference of each variant is different, but the difference in the epitope score caused by a single mutation is not obvious enough. It may require multiple mutations to cooperate to achieve the immune escape of the virus. Furthermore, by pseudovirus system, flow cytometry, and surface plasmon resonance analysis, we found that for a single S protein, the infectivity of Omicron S protein and its binding ability to ACE2 were significantly reduced as compared with other variants. To truly reflect the virus infection, we infected Vero cells with authentic viruses. Then, the infectivity, replication ability, and cytotoxicity of the virus were analyzed. Our results suggest that Omicron possesses lower infectivity and cytotoxicity than other strains. However, more animal experiments are still needed to characterize the biological property of Omicron in vivo.

At the same time, its replication ability is also lower than that of the Delta variant. Our results suggest that Omicron possesses lower infectivity and cytotoxicity than other variants, and its replication capacity is also lower than that of Delta variant.

Since the first coronavirus case was reported in December 2019 and the first sequence of SARS‐CoV‐2 was announced, the virus has mutated multiple times. Although RNA viruses may undergo natural genetic attenuation, these viruses may cause the transient nature of the pandemic.^26^ Analysis of mutations based on isolates in different regions indicates that the virus may have spread globally before the pandemic.^27^ In July 2020, a strain with the spike protein D614G mutation was discovered in Europe and subsequently became the main form of the virus pandemic.^4^ Recently, B.1.617.2 (Delta variant) and B1.1.529 (Omicron) have spread worldwide at a higher speed.^6^, ^7^ It has been reported that SARS‐CoV‐2 is still in the rapid evolution stage. Static equilibrium has not been reached yet. Although there are many nonsynonymous mutations in the genome, the viral genome is in a state of negative selection.^28^ The SARS‐CoV‐2 S protein has three hinge‐like structures.^24^ This structure makes the S protein flexible and abnormal. Under this structure, S protein can swing and rotate to help scan a larger area of the cell surface and help bind to the ACE2 receptor. SARS‐CoV also binds to ACE2, but the binding strength of the new coronavirus is two to four times higher,^29^ because CoV‐2 has more survival‐suitable mutations in the S protein. Alpha, Delta, and Omicron P681 mutations will reduce the acidity of the amino acid sequence, thereby increasing the efficiency of furin recognition and cleavage.^25^ This increases the efficiency of the virus infecting host cells.

Mutations in the spike protein of some SARS‐CoV‐2 variants can cause immune escape responses. For example, E484K mutations are currently known to affect the neutralization of serum or monoclonal antibodies (mAb) during the recovery period. Similarly, the combined mutation of K417N and N501Y affects the neutralization of mAb and convalescent serum.^30^ The same spike proteins, K444E, G446 V, L452R, and F490S, can also evade serum neutralization. S477G, S477N, and S477R occupy a prominent position in mAb escape mutations.^31^ A study identified several repeated deletion regions (RDRs) in the NTD and found that Δ69‐70 and Δ243‐244 can eliminate the binding of neutralizing antibodies.^31^ In the Alpha and Omicron strains, two key deletions, H60V70, are on the epitope, which can affect the immune escape of the S protein (Figure 4F), whereas N501Y and D614G have almost no effect on the antigen score, and the P681 mutation slightly reduces the epitope score and may have a certain impact. Studies have shown that compared with the farthest strains Beta and Delta before, Omicron is farther away from the original SARS‐CoV‐2 vaccine strain on the antigen. There is clear evidence that the antigenicity of the spike protein of SARS‐CoV‐2 has changed and the amino acid changes that affect antibody neutralization. Amino acid mutations and deletions of spike proteins that affect neutralizing antibodies are present in different variants around the world with significant frequency, and there is new evidence that virus variants are resistant to vaccine‐induced antiviral responses.^32^ Overall, we did not find significant changes in the epitope scores in these variants. It is possible that the neutralizing antibody escape of the variants is more caused by their conformational changes. It has been reported that the basic infection rate of the Delta variants is R0; that is, the number of infections per infected person is as high as eight to nine people, and it is more likely to cause severe illness. The prototype strain of the new coronavirus has an R0 value of 2, which is equivalent to a common cold.^33^ Studies have shown that compared with the farthest strains Beta and Delta before, Omicron is farther away from the original SARS‐CoV‐2 vaccine strain on the antigen. And the neutralizing antibody experiment has also proved that the Omicron strain has a stronger immune escape ability.^34^ Although there are various forms of vaccines against SARS‐CoV‐2, the vaccines against Omicron variant are not satisfactory so far. The bivalent vaccine prepared by the combination of S1‐WT and S1‐Mut proteins, developed by He Cai's team, showed cross‐protection against wild‐type and SARS‐CoV‐2 variants.^35^ This may provide new ideas for the development of vaccines against SARS‐CoV‐2 variants in the future.

In the Omicron variant, there are some consistent RBD mutations with previously focused variants (K417N, T478K, and N501Y). The N501Y and K417N mutations confer increased and decreased binding affinity to ACE2, respectively.^21^ These mutations did not significantly alter affinity for ACE2 when present alone or in combination with other RBD mutations.^21^ Most of the mutations have been shown to reduce Omicron receptor binding, except for G339D, N440K, S447N, and Q498R.^22^, ^23^ However, some new mutations in Omicron strains have strengthened the effects on ACE2 affinity, such as new mutations at residues 493, 496, and 501, which restore the Omicron receptor binding ability to some extent.^21^ It has been found that the serum neutralizing activity against Omicron produced after vaccination is significantly reduced, but the serum neutralizing ability can be basically restored after the third dose of vaccine. Most receptor‐binding motif‐directed monoclonal antibodies lost their in vitro neutralizing activity against Omicron.^36^

The binding affinity of the Omicron RBD region to ACE2 has not been reported to be significantly increased, but the ability of the pseudovirus to enter HEK293T‐ACE2 cells is indeed enhanced by the highly mutated spike.^37^ Also compared with other SARS‐CoV‐2 VOCs, the Omicron VOC shows the enhanced binding to the ACE2 orthologs of human and mouse,^36^ which contradicts our results. However, some researchers have shown by computer simulation and ELISA experiments that the Omicron variant has comparable binding ability to human ACE2 compared with wild‐type SARS‐CoV‐2, but much weaker binding affinity than the Delta variant,^38^ which is consistent with the results in this study. However, these results are based on pseudovirus systems or in vitro binding experiments, which cannot reflect the real situation of virus infection. In contrast, infection experiments of VeroE6/TMPRSS2 cells demonstrated that the Omicron variant showed weaker cell fusion activity compared to the Delta variant.^39^ Similar results were obtained from infection experiments of authentic viruses on Vero cells. Omicron appears to be weaker than Delta and other variants in terms of infectivity on Vero cells, and second to Delta in viral replication. These results support the reliability of our pseudovirus results. It may be due to the numerous mutations of Omicron S protein that affect its binding ability to ACE2.

As viral infection is also affected by many other factors besides receptor binding, the impact of these findings on the ability of the virus to infect and transmit in humans remains to be assessed. There are indications that Omicron variants appear to have evolved to selectively balance the ability to increase immune evasion with the ability to maintain potent ACE2 binding. Recently, it was found that the pathogenicity and replication ability of Omicron strains were weakened compared with other variants.^40^ We also observed this in Vero cells. But whether this means that SARS‐CoV‐2 is undergoing natural attenuation, and whether the impact of SARS‐CoV‐2 on human health will gradually weaken in the future, these still need further research.

## 4 MATERIALS AND METHODS

### 4.1 Viral genome sources

The virus genomes involved in this research are all derived from NCBI, prototype (NC 045512), Alpha (OM486838.1), Beta (OM463433.1), Delta (OM432946.1), Omicron (OV647874.1), and genome comparison and mutation analysis are completed by SnapGene software.

### 4.2 S protein tertiary structure and B‐cell epitope prediction

Through the Swiss‐Mode online platform, the tertiary structure of the spike protein of the five strains was preliminarily entered. After adding the complete amino acid sequence of the S protein in the text box, click to establish the model, and wait for the system to complete the model prediction and output. Then, the model with the highest coverage rate is selected as the final model according to the multiple prediction versions given. The spike protein was rendered and annotated by PyMOL software, and the mutation sites were highlighted as green dots. B‐cell epitope prediction and scoring of spike proteins was done by the IEBD online program (https://www.iedb.org/). In short, select Antigen Sequence Properties under B Cell Epitope Prediction on the IEBD website, then enter the complete amino acid sequence of S protein and select the Bepipred Linear Epitope Prediction 2.0 program to start predicting B‐cell epitopes. After the prediction is completed, set the threshold to 0.5. Analyze the antigenicity of different sites.

### 4.3 Pseudovirus infectivity test

For pseudovirus infection experiments, the prototype, Alpha, Beta, Delta, and Omicron pseudoviruses with luciferase expressing were purchased from Genomeditech (Prototype GM‐0220PV07; Alpha GM‐0220PV33; Beta GM‐0220PV32; Delta GM‐0220PV45; Omicron GM‐0220PV84, Shanghai, China). The same amount of SARS‐CoV‐2 VOCs pseudovirus was used in each experiment, and 100 μl of 4 × 10^3^ TU of SARS‐CoV‐2 VOCs pseudoviruses was added to each well of an opaque 96‐well plate in the experiment, and then 1.2 × 10^4^ 293T‐ACE2 cells were added. Plates were then incubated for 48 h in a 5% CO_2_ incubator at 37°C. Finally, the cell supernatant was removed, and 100 μl lysis reagent with luciferase substrate (Promega, USA) was added to determine the relative light unit (RLU) by a multimode microplate reader (PerkinElmer, USA). Fifty percent of the neutralizing titers of pseudovirus was expressed as the highest dilution that caused 50% inhibition relative to the average of the virus control wells and calculated by a nonlinear regression model (inhibitor vs. normalized response) in GraphPad 8.0 software.

### 4.4 Flow cytometric analysis

RBD‐hFc protein (Prototype RBD SPD‐C5255; Alpha RBD, SPD‐C5253; Beta RBD SPD‐C5256; Delta RBD, SPD‐C525d, purchased from ACRO Biosystems; Omicron RBD, 40592‐V05H3 Sino Biological, purchased from Sino Biological) was diluted to 1 μg/ml with PBS buffer, then each concentration gradient was diluted twice; total of eight concentration gradients were set. 293T‐ACE2 cells were collected into flow tubes (2.5 × 10^5^ cells/tube). After washing once with PBS, the liquid was poured out for use. Add 100 μl of the RBD diluent in the previous step to the flow tube, respectively, and incubate at 37°C for 40 min. Add 2 ml of PBS to wash unbound proteins, and add 1 μl of Fc Tag flow antibody (BioLegend USA) to each tube for staining at 4°C for 30 min. The excess antibody was washed with PBS, and 500 μl of PBS solution was added for detection on the machine.

### 4.5 RBD and ACE2 affinity test

Surface plasmon resonance analysis was used to detect and analyze biomolecular interactions. Surface plasmon resonance (SPR)‐based measurements were performed by Biacore 8K (GE Healthcare), as previously described.^11^ One hundred response units (RUs) of human ACE2–Fc (AC2‐H5257) were captured on Sensor Chip Protein A. For kinetic analysis, RBD protein was run across the chip in a two‐fold dilution series (1, 2, 4, 8, 16, 32 nM), with one channel set as control. Each sample that was bound to the antigen surface was dissociated by HBS‐EP + running buffer for 300 s at a flow rate of 30 μl/min. Regeneration of the sensor chips was performed for 60 s using regeneration buffer (glycine pH 1.5). The affinity value (dissociation constant *K*_d_) was determined. Higher affinity value (*K*_d_) means lower the affinity with ACE2.

### 4.6 Virus infection experiment

The SARS‐CoV‐2 VOCs were provided by the National Kunming High‐level Biosafety Primate Research Center. The prototypic SARS‐CoV‐2 strain and Beta variant were gifted by Guangdong CDC, Alpha variant from China CDC, Delta variant from Chongqing CDC, and Omicron variant from Institute of Laboratory Animal Sciences, CAMS & PUMC. The virus infection experiment was conducted in the BSL3/4 laboratory, and all operations follow the corresponding regulations of biosafety laboratory. Prototype SARS‐CoV‐2 and VOCs were used to infect Vero cells. Briefly, cells were seeded into 24‐well (2 × 10^5^ cells/well) or 12‐well (2 × 10^5^ cells/well) plates at 12 h before infection. Viruses at the multiplicity of infection (MOI) of 0.05 were added to the cells, followed by incubation at room temperature for 1 h. Then the unbound viruses in the supernatant were removed, and maintenance medium (DMEM containing 2% serum) was added to each well for continuous culture in a 37°C 5% CO_2_ incubator.

### 4.7 qPCR test

The cells and supernatants in the 24‐well plate were harvested at 2, 24, 48, and 72 h after infection, and then frozen and thawed once at −80°C, followed by centrifugation at 1000 rpm for 5 min. Then, 200 μl of the supernatant was transferred to a new tube with addition of 600 μl Trizol to lyze viruses. RNA extraction was performed using KingFisher Flex Purification System (ThermoFisher) automatic nucleic acid extraction instrument and MagMAX‐96 nucleic acid extraction kit (ThermoFisher AM1836). The operation was performed according to the reagent manufacturer's instructions. TaqMan Fast Viral 1‐Step Master Mix (ThermoFisher, 4444432) was used for qPCR. Copy number of viral genomes was measured with N gene, and subgenome copy number analyzed by E‐gene. In short, 2.5 μl PCR mix was added, 0.5 μl upstream and downstream primers (gRNA‐N‐F: GACCCCAAAATCAGCGAAAT; gRNA‐N‐R: TCTGGTTACTGCCAGTTGAATCTG) (sgRNA‐E‐F: CGATCTCTTGTAGATCTGTTCTC; sgRNA‐E‐R: ATATTGCAGCAGTACGCACACA), 0.5 μl probe (gRNA‐N: FAM‐ACCCCGCATTACGTTTGGTGGACC‐BHQ1) (sgRNA‐E‐FAM: ACACTAGCCATCCTTACTGCGCTTCG‐BHQ1), 3.5 μl H_2_O, and 2.5 μl RNA template to each well of a 384‐well plate. The reaction was then performed on a CFX384 Touch fluorescence quantitative PCR instrument (Bio‐Rad USA). After the reaction was completed, the data were collected, and the virus copy number was calculated according to the standard.

### 4.8 Immunofluorescence analysis

The cells in the 12‐well plate were washed three times with PBS at 2 and 24 h after infection, and then fixed with 4% paraformaldehyde for 30 min. Cells was permeabilized with PBST (0.1% Triton × 100) for 20 min and washed three times with PBS. Cells were then blocked with 3% goat serum for 1 h and washed three times with PBST. Then, 200 μl of N protein antibody (rabbit antibody, SinoBiological) diluted at 1:500 was added to cells, followed by incubation at 4°C overnight. Next day, cells were washed three times with PBST. Goat anti‐rabbit secondary antibody (Invitrogen A‐11020) diluted at 1:5000 was added to cells, followed by incubation for 2 h at room temperature. Cells were washed three times with PBS. The slides were mounted with anti‐fade mounting medium ProLong Gold (ThermoFisher P10144) (with DAPI) and finally imaged under a Leica TCS SP8 CARS (Leica) confocal microscope.

## CONFLICT OF INTEREST

The authors declare that there is no conflict of interest.

## ETHICS STATEMENT

Not applicable.

## AUTHOR CONTRIBUTIONS

Shuaiyao Lu and Xiaozhong Peng provided study concepts and designed the study. Hao Yang, Penghui Liu and Yanan Zhou completed the main experiments and analysis of the results. Yong Zhang and Tingfu Du provided guidance on experimental techniques. Shuaiyao Lu and Hao Yang wrote the manuscript.

## Supporting information

Supporting Information

## Data Availability

The data included in this study are available upon request from the corresponding author.

## References

[1] WHO . *Coronavirus (COVID‐19) Pandemic*. WHO; 2022. Accessed February 7, 2022. https://covid19.who.int/

[2] Rocklöv J , Sjödin H , Wilder‐Smith A . COVID‐19 outbreak on the Diamond Princess cruise ship: estimating the epidemic potential and effectiveness of public health countermeasures. J Travel Med. 2020;27(3):taaa030. 32109273 10.1093/jtm/taaa030 PMC7107563

[3] Islam KU , Iqbal J . An update on molecular diagnostics for COVID‐19. Front Cell Infect Microbiol. 2020;10:560616. 33244462 10.3389/fcimb.2020.560616 PMC7683783

[4] Korber B , Fischer WM , Gnanakaran S , et al. Tracking changes in SARS‐CoV‐2 spike: evidence that D614G increases infectivity of the COVID‐19 virus. Cell. 2020;182(4):812‐827. 32697968 10.1016/j.cell.2020.06.043 PMC7332439

[5] Funk T , Pharris A , Spiteri G , et al. Characteristics of SARS‐CoV‐2 variants of concern B.1.1.7, B.1.351 or P.1: data from seven EU/EEA countries, weeks 38/2020 to 10/2021. Eurosurveillance. 2021;26(16):2100348. 10.2807/1560-7917.ES.2021.26.16.2100348 PMC8063589 33890566

[6] Yadav PD , Sapkal GN , Abraham P , et al. Neutralization potential of Covishield vaccinated individuals sera against B.1.617.1. Clin Infect Dis. 2021;74(3):558‐559. 10.1093/cid/ciab483 34036309

[7] Kandeel M , Mohamed MEM , Abd El‐Lateef HM , Venugopala KN , El‐Beltagi HS . Omicron variant genome evolution and phylogenetics. J Med Virol. 2021;94(4):1627‐1632. 34888894 10.1002/jmv.27515 PMC9015349

[8] Chen J , Wang R , Gilby NB , Wei GW . Omicron variant (B.1.1.529): infectivity, vaccine breakthrough, and antibody resistance. J Chem Inf Model. 2022;62(2):412‐422. 34989238 10.1021/acs.jcim.1c01451 PMC8751645

[9] Kadam SB , Sukhramani GS , Bishnoi P , Pable AA , Barvkar VT . SARS‐CoV‐2, the pandemic coronavirus: molecular and structural insights. J Basic Microbiol. 2021;61(3):180‐202. 33460172 10.1002/jobm.202000537 PMC8013332

[10] de Haan CA , Kuo L , Masters PS , Vennema H , Rottier PJ . Coronavirus particle assembly: primary structure requirements of the membrane protein. J Virol. 1998;72(8):6838‐6850. 9658133 10.1128/jvi.72.8.6838-6850.1998 PMC109893

[11] Wrapp D , Wang N , Corbett KS , et al. Cryo‐EM structure of the 2019‐nCoV spike in the prefusion conformation. Science. 2020;367(6483):1260‐1263. 32075877 10.1126/science.abb2507 PMC7164637

[12] Xu X , Chen P , Wang J , et al. Evolution of the novel coronavirus from the ongoing Wuhan outbreak and modeling of its spike protein for risk of human transmission. Sci China Life Sci. 2020;63(3):457‐460. 32009228 10.1007/s11427-020-1637-5 PMC7089049

[13] Lan J , Ge J , Yu J , et al. Structure of the SARS‐CoV‐2 spike receptor‐binding domain bound to the ACE2 receptor. Nature. 2020;581(7807):215‐220. 32225176 10.1038/s41586-020-2180-5

[14] Liu H , Wei P , Kappler JW , Marrack P , Zhang G . SARS‐CoV‐2 variants of concern and variants of interest receptor binding domain mutations and virus infectivity. Front Immunol. 2022;13:825256. 35154144 10.3389/fimmu.2022.825256 PMC8828474

[15] Yurkovetskiy L , Wang X , Pascal KE , et al. Structural and functional analysis of the d614g sars‐cov‐2 spike protein variant. Cell. 2020;183(3):739‐751.[crossref] 32991842 10.1016/j.cell.2020.09.032 PMC7492024

[16] Zhang J , Cai Y , Xiao T , et al. Structural impact on SARS‐CoV‐2 spike protein by D614G substitution. Science. 2021;372(6541):525‐530. 33727252 10.1126/science.abf2303 PMC8139424

[17] Jiang X , Zhang Z , Wang C , et al. Bimodular effects of D614G mutation on the spike glycoprotein of SARS‐CoV‐2 enhance protein processing, membrane fusion, and viral infectivity. Signal Transduct Target Ther. 2020;5(1):268. 33203835 10.1038/s41392-020-00392-4 PMC7670837

[18] Mohammad A , Alshawaf E , Marafie SK , Abu‐Farha M , Abubaker J , Al‐Mulla F . Higher binding affinity of furin for SARS‐CoV‐2 spike (S) protein D614G mutant could be associated with higher SARS‐CoV‐2 infectivity. Int J Infect Dis. 2021;103:611‐616. 33075532 10.1016/j.ijid.2020.10.033 PMC7567667

[19] Guglielmi G . Rapid coronavirus tests: a guide for the perplexed. Nature. 2021;590(7845):202‐205. 33564189 10.1038/d41586-021-00332-4

[20] Cai Y , Zhang J , Xiao T , et al. Structural basis for enhanced infectivity and immune evasion of SARS‐CoV‐2 variants. Science. 2021;373(6555):642‐648. 34168070 10.1126/science.abi9745 PMC9245151

[21] Mannar D , Saville JW , Zhu X , et al. SARS‐CoV‐2 Omicron variant: antibody evasion and cryo‐EM structure of spike protein‐ACE2 complex. Science. 2022;375(6582):760‐764. 35050643 10.1126/science.abn7760 PMC9799367

[22] Starr TN , Greaney AJ , Hilton SK , et al. Deep mutational scanning of SARS‐CoV‐2 receptor binding domain reveals constraints on folding and ACE2 binding. Cell. 2020;182(5):1295‐1310.e20. 32841599 10.1016/j.cell.2020.08.012 PMC7418704

[23] Zahradník J , Marciano S , Shemesh M , et al. SARS‐CoV‐2 variant prediction and antiviral drug design are enabled by RBD in vitro evolution. Nat Microbiol. 2021;6(9):1188‐1198. 34400835 10.1038/s41564-021-00954-4

[24] Turoňová B , Sikora M , Schürmann C , et al. In situ structural analysis of SARS‐CoV‐2 spike reveals flexibility mediated by three hinges. Science. 2020;370(6513):203‐208. 32817270 10.1126/science.abd5223 PMC7665311

[25] Scudellari M . How the pandemic might play out in 2021 and beyond. Nature. 2020;584(7819):22‐25. 32760050 10.1038/d41586-020-02278-5

[26] Brewer WHSF , Sanford JC . Information loss: potential for accelerating natural genetic attenuation of RNA viruses. Biol Inform. 2013:369‐384. 10.1142/9789814508728_0015

[27] van Dorp L , Acman M , Richard D , et al. Emergence of genomic diversity and recurrent mutations in SARS‐CoV‐2. Infect Genet Evol. 2020;83:104351. 32387564 10.1016/j.meegid.2020.104351 PMC7199730

[28] Yi K , Kim SY , Bleazard T , Kim T , Youk J , Ju YS . Mutational spectrum of SARS‐CoV‐2 during the global pandemic. Exp Mol Med. 2021;53(8):1229‐1237. 34453107 10.1038/s12276-021-00658-z PMC8393781

[29] Nguyen HL , Lan PD , Thai NQ , Nissley DA , O'Brien EP , Li MS . Does SARS‐CoV‐2 bind to human ACE2 more strongly than does SARS‐CoV? J Phys Chem B. 2020;124(34):7336‐7347. 32790406 10.1021/acs.jpcb.0c04511 PMC7433338

[30] Collier DA , De Marco A , Ferreira I , et al. Sensitivity of SARS‐CoV‐2 B.1.1.7 to mRNA vaccine‐elicited antibodies. Nature. 2021;593(7857):136‐141. 33706364 10.1038/s41586-021-03412-7 PMC7616976

[31] Liu Z , VanBlargan LA , Bloyet LM , et al. Identification of SARS‐CoV‐2 spike mutations that attenuate monoclonal and serum antibody neutralization. Cell Host Microbe. 2021;29(3):477‐488. 33535027 10.1016/j.chom.2021.01.014 PMC7839837

[32] Harvey WT , Carabelli AM , Jackson B , et al. SARS‐CoV‐2 variants, spike mutations and immune escape. Nat Rev Microbiol. 2021;19(7):409‐424. 34075212 10.1038/s41579-021-00573-0 PMC8167834

[33] Brown CM , Vostok J , Johnson H , et al. Outbreak of SARS‐CoV‐2 infections, including COVID‐19 vaccine breakthrough infections, associated with large public gatherings ‐ Barnstable County, Massachusetts, July 2021. MMWR Morb Mortal Wkly Rep. 2021;70(31):1059‐1062. 34351882 10.15585/mmwr.mm7031e2 PMC8367314

[34] Dejnirattisai W , Shaw RH , Supasa P , et al. Reduced neutralisation of SARS‐CoV‐2 Omicron B.1.1.529 variant by post‐immunisation serum. Lancet. 2022;399(10321):234‐236. 34942101 10.1016/S0140-6736(21)02844-0 PMC8687667

[35] Wang J , et al. A bivalent recombinant vaccine: a promising strategy against both SARS‐CoV‐2 variants and wild type of the virus. Signal Transduct Target Ther. 2021;6(1):278. 34274941 10.1038/s41392-021-00691-4 PMC8285695

[36] Cameroni E , Bowen JE , Rosen LE , et al. Broadly neutralizing antibodies overcome SARS‐CoV‐2 Omicron antigenic shift. Nature. 2022;602(7898):664‐670. 35016195 10.1038/s41586-021-04386-2 PMC9531318

[37] Zhang X , Wu S , Wu B , et al. SARS‐CoV‐2 Omicron strain exhibits potent capabilities for immune evasion and viral entrance. Signal Transduct Target Ther. 2021;6(1):430. 34921135 10.1038/s41392-021-00852-5 PMC8678971

[38] Wu L , Zhou L , Mo M , et al. SARS‐CoV‐2 Omicron RBD shows weaker binding affinity than the currently dominant Delta variant to human ACE2. Signal Transduct Target Ther. 2022;7(1):8. 34987150 10.1038/s41392-021-00863-2 PMC8727475

[39] Zhao H , Lu L , Peng Z , et al. SARS‐CoV‐2 Omicron variant shows less efficient replication and fusion activity when compared with Delta variant in TMPRSS2‐expressed cells. Emerg Microbes Infect. 2022;11(1):277‐283. 34951565 10.1080/22221751.2021.2023329 PMC8774049

[40] Shuai H , Chan JF , Hu B , et al. Attenuated replication and pathogenicity of SARS‐CoV‐2 B.1.1.529 Omicron. Nature. 2022;7902(1):693‐699. 10.1038/s41586-022-04442-5 35062016

