# Supplementary material for: Characteristic analysis of Omicron‐included SARS‐CoV‐2 variants of concern
Source: MedComm (2020). 2022 Apr 9;3(2):e129. doi: 10.1002/mco2.129 (PMC8994548; doi:10.1002/mco2.129)
Supplement: Supplementary file 1 — Supporting Information [file MCO2-3-0-s001.docx]

**Supplementary materials for**

**Characteristic analysis of Omicron-included SARS-CoV-2 variants of concern**

Hao Yang^1#^, Penghui Liu^1#^, Yong Zhang^1^, Tingfu Du^1^, Yanan Zhou^1^, Shuaiyao Lu^1*^, Xiaozhong Peng^1,2*^

1Institute of Medical Biology, Chinese Academy of Medical Sciences and Peking Union Medical College, Kunming, Yunnan China

2State Key Laboratory of Medical Molecular Biology, Department of Molecular Biology and Biochemistry, Institute of Basic Medical Sciences, Medical Primate Research Center, Neuroscience Center, Chinese Academy of Medical Sciences, School of Basic Medicine Peking Union Medical College, Beijing, China

1National Kunming High-Level Biosafety Primate Research Center, Institute of Medical Biology, Chinese Academy of Medical Sciences and Peking Union Medical College, Kunming, Yunnan China

2State Key Laboratory of Medical Molecular Biology, Department of Molecular Biology and Biochemistry, Institute of Basic Medical Sciences, Medical Primate Research Center, Neuroscience Center, Chinese Academy of Medical Sciences, School of Basic Medicine Peking Union Medical College, Beijing, China

**
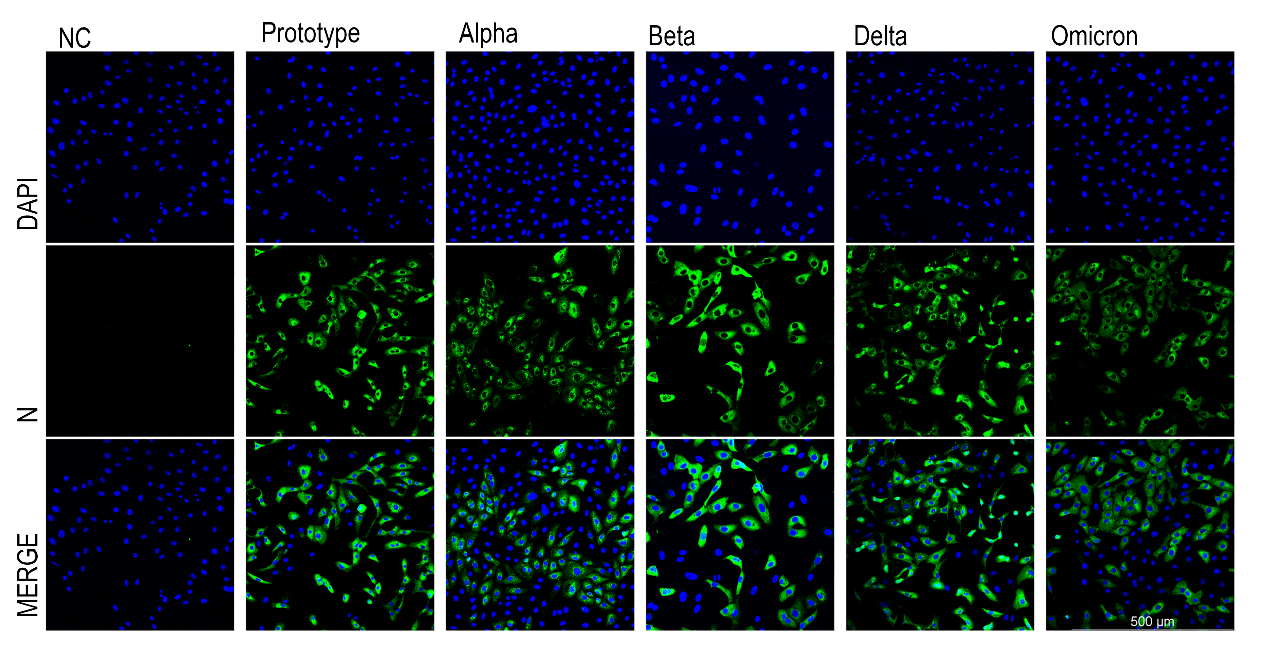
**

**Supplementary Figure 1** Immunofluorescence analysis of viral antigen of different variants in Vero cells 24 h after virus infection. Viral antigen was detected with anti-N protein antibody. Shot under a 20x confocal microscope.

**
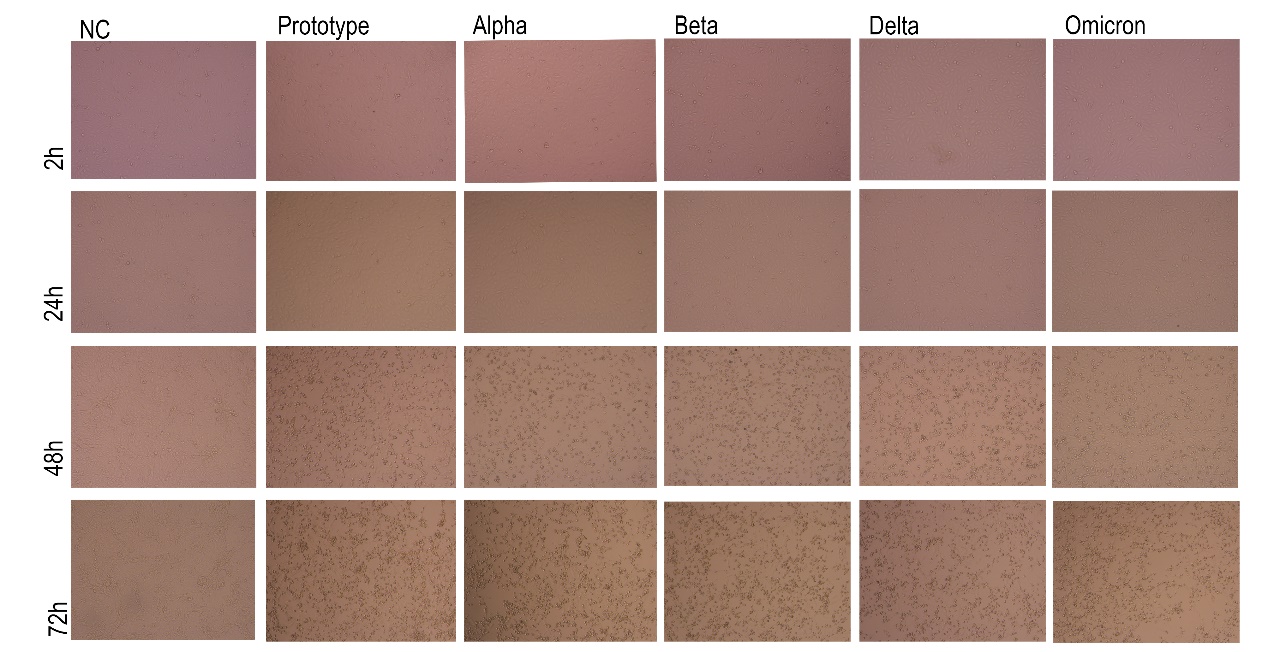
**

**Supplementary Figure 2** The CPE of Vero cells at 2, 24, 48 and 72 hours after virus infection was photographed under a 20x microscope.
